# Screen-printed Microsensors Using Polyoctyl-thiophene (POT) Conducting Polymer As Solid Transducer for Ultratrace Determination of Azides

**DOI:** 10.3390/molecules24071392

**Published:** 2019-04-09

**Authors:** Ahmed Galal Eldin, Abd El-Galil E. Amr, Ayman H. Kamel, Saad S. M. Hassan

**Affiliations:** 1 Chemistry Department, Faculty of Science, Ain Shams University, 11566 Abbasia, Cairo, Egypt; ahmeddna2006@yahoo.com; 2 Pharmaceutical Chemistry Department, Drug Exploration & Development Chair (DEDC), College of Pharmacy, King Saud University, Riyadh 11451, Saudi Arabia; 3 Applied Organic Chemistry Department, National Research Centre, 12622 Dokki, Giza, Egypt

**Keywords:** azides, screen-printed sensors, poly (3-octylthiophene), solid contact potentiometric sensors, iron-phthalocyanine, nitron-azide complexes

## Abstract

Two novel all-solid-state potentiometric sensors for the determination of azide ion are prepared and described here for the first time. The sensors are based on the use of iron II-phthalocyanine (Fe-PC) neutral carrier complex and nitron-azide ion-pair complex (Nit-N_3_^−^) as active recognition selective receptors, tetradodecylammonium tetrakis(4-chlorophenyl) borate (ETH 500) as lipophilic cationic additives and poly(octylthiophene) (POT) as the solid contact material on carbon screen-printed devices made from a ceramic substrate. The solid-contact material (POT) is placed on a carbon substrate (2 mm diameter) by drop-casting, followed, after drying, by coating with a plasticized PVC membrane containing the recognition sensing complexes. Over the pH range 6-9, the sensors display fast (< 10 s), linear potentiometric response for 1.0 × 10^−2^–1.0 × 10^−7^ M azide with low detection limit of 1.0 × 10^−7^ and 7.7 × 10^−8^ M (i.e., 6.2–4.8 ng/ml) for Fe-PC/POT/and Nit-N_3_^−^/POT based sensors, respectively. The high potential stability and sensitivity of the proposed sensors are confirmed by electrochemical impedance spectroscopy (EIS) and constant-current chronopotentiometry measurement techniques. Strong membrane adhesion and absence of delamination of the membrane, due to possible formation of a water film between the recognition membranes and the electron conductor are also verified. The proposed sensors are successfully applied for azide quantification in synthetic primer mixture samples. Advantages offered by these sensors are the robustness, ease of fabrication, simple operation, stable potential response, high selectivity, good sensitivity and low cost.

## 1. Introduction

Coated wire potentiometric electrodes (CWEs) are prepared by direct coating of metallic conductors such as platinum, copper, silver, gold and carbon substrates with electroactive species incorporated in a thin polymeric film. These devices are simple, inexpensive, do not need internal reference solutions, are easily miniaturized, and find application in chemical, biomedical and clinical analyses. The development of chemical field-effect transistors (CHEMFETs) was considered a logical extension of these devices [1,2]. However, these sensors gave low potential stability and poor reproducibility due to the direct contact betwwen two phases of quite different properties. The observed instability is attributed to the formation of a thin electrolyte solution layer between the conductive support and the membrane with composition changing in course of the measurement [3].

Recently, solid-contact potentiometric ion-selective sensors (SC-ISEs) based on the use of conducting polymers (CPs) have received considerable attention [4,5] and demonstrated a wide range of applications. Among the many conducting polymers that have been investigated, polypyrrole, poly(3-octylthiophene) (POT), polyaniline, and poly(3,4-ethylenedioxythiophene) (PEDOT) are the most commonly used polymers [4,5]. POT is less subject to reactions with ambient species such as oxygen because it has a relatively high oxidation potential and is usually used in an undoped ion-free form. Thus POT films have a relatively low redox capacitance and electronic conductivity. Ion-to-electron transduction at the interfaces occurs mainly through the electrical double layer that forms at the solid contact/membrane boundary [5].

This configuration seems ideal for fabrication of miniaturized sensors with sufficient analytical performance for trace analysis of some metal ions [6,7,8,9]. Unlike the conventional designs of potentiometric sensors, solid contact sensors are compatible with mass production technologies, which drive down unit cost and improving batch- to - batch reproducibility. On the other hand, the screen-printing technique has been successfully applied to mass production of low-cost, highly reproducible and reliable disposable sensors for rapid assessment of many types of analytes [10,11,12].

The present study deals with fabrication and application of solid contact potentiometric sensors with conducting polymer for sensitive and selective determination of azides which are widely used in explosive detonators, electrical discharge tubes, anti-corrosion solutions, production of foam rubber, laboratories preservatives, agricultural pest control, and automobile air bags [13]. On the other hand, azides are considered as potent toxins, similar to cyanides as both cause death due strongly binding to iron in hemoglobin [14]. An intake of about 10 µg/g azide ions causes death within half an hour [15]. In addition, it is readily protonated in the aquatic environment to yield volatile hydrazoic acid (HN_3_) that can create an airborne hazard [16]. Therefore, tight controls on the allowed levels of azides in wastewater effluents, industrial solid waste and propellants are highly demanded.

Few azide-potentiometric polymeric membrane and gas sensors have been described [17,18,19,20,21,22,23,24,25,26]. Some of these devices exhibit narrow working concentration ranges and suffer from interference from various anions such as ClO_4_^−^, SO_4_^2−^, HCO_3_^−^, Cl^−^, HPO_4_^2−^, and NO_3_^−^. In order to cope with these limitations, further efforts are required to develop new designs of potentiometric probes with lower detection limits, good selectivity, and high potential stability for azide monitoring.

In this work, novel solid contact potentiometric sensors using carbon screen-printed substrates were prepared, optimized and examined. These sensors are based on the use of Fe^II^PC ionophore and Nit-N_3_^−^ ion association complex as sensing materials for azide ions. These sensors are applicable for trace analysis of azide ion. The performance characteristics of these sensors were evaluated and satisfactorily used for accurate determination of microgram quantities of azide. The sensors offered excellent advantages such as miniaturization, cost-effectiveness, ease of fabrication and high potential stability and sensitivity.

## 2. Results

### 2.1. Sensors Construction and Characteristics

**Figure 1:**
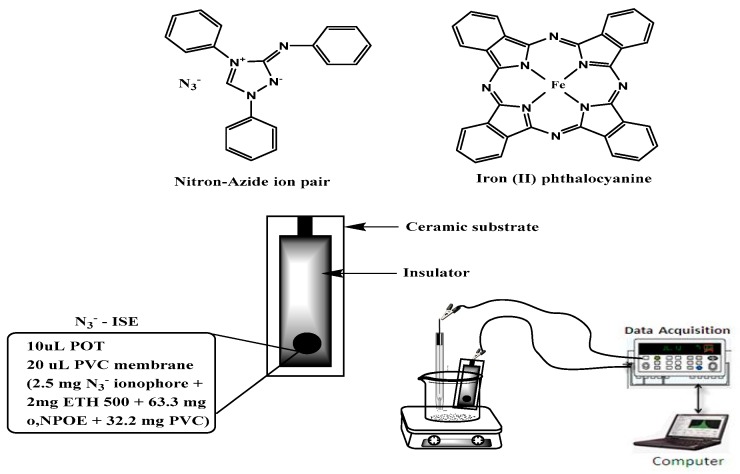
Chemical structures of azide ionophores used for azide membrane sensors and a schematic presentation of the proposed device.

Two all-solid state potentiometric microsensors were prepared, characterized and evaluated. These consist of a carbon screen printed planar ceramic substrate (2 × 2 mm) coated with poly- (octylthiophene) (POT) as a solid conducting layer and covered with a film of either iron(II)-phthalocyanine complex (FePC) or nitron-azide ion-pair complex (Nit-N_3_^−^) dispersed in the plasticized PVC film as active recognition selective receptors (Figure 1). The sensor cocktail consists of PVC, sensing ionophore, ETH 500 and the plasticizer in an optimum ratio of 32.2, 2.5, 2.0 and 63.3 wt%, respectively. ETH 500 was used as ion excluder and *o*-NPOE as a membrane plasticizer [27,28]. The plasticizer was selected to possess high lipophilicity, high molecular weight, low tendency for exudation from the membrane matrix, low vapor pressure and high capacity to dissolve the membrane ingradients. The use of ETH 500 additive in the membrane provided significant effect on the sensor response [29]. *o*-NPOE (*Ɛ_r_* = 24, M.wt. 435), DBS (*Ɛ_r_* = 5.1, M.wt. 390), and DOP (*Ɛ_r_* = 8.4, M.wt. 390.5 plasticizers were tested.

**Figure 2:**
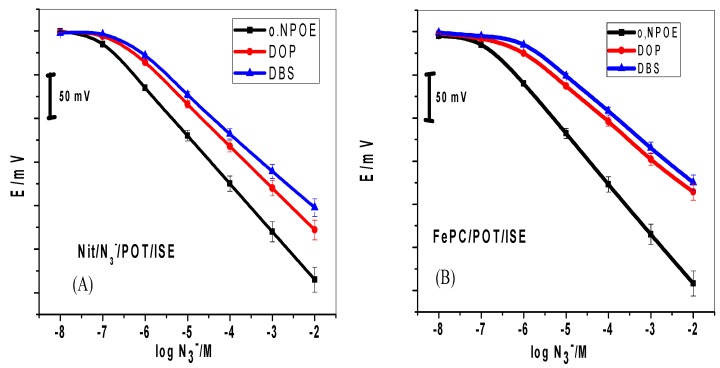
Effect of plasticizer polarity on the potentiometric plot of (**A**) Nit/N_3_^−^/POT-ISE and (**B**) FePC/POT-ISE. Background: 10^−2^ M Tris buffer solution, pH 7.0.

The calibration plots of membrane sensors containing these solvent mediators are shown in Figure 2. Linear responses for the concentration ranges of 3.5 × 10^−7^–1.0 × 10^−2^, 1.0 × 10^−6^–1.0 × 10^−2^ and 2.6 × 10^−6^–1.0 × 10^−2^ M, and detection limits of 1.0 × 10^−7^, 4.3 × 10^−7^ and 7.2 × 10^−7^ M with calibration slopes of −58.3 ± 0.9, −41.3 ± 0.6 and −41.4 ± 0.2 mV/decade for were obtained with FePC/POT based membranes plasticized with *o*-NPOE, DOP and DBS, respectively. Nit-N_3_/POT based membrane sensors plasticized with *o*-NPOE, DOP and DBS, displayed calibration slopes of −55.1 ± 0.7, 48.2 ± 0.6 and −43.6 ± 0.5 mV/decade, linear responses over the concentration ranges of 1.0 × 10^−7^–1.0 × 10^−2^, 1.0 × 10^−6^–1.0 × 10^−2^ and 1.0 × 10^−6^–1.0 × 10^−2^ M, and detection limits 7.7 × 10^−8^, 2.1 × 10^−7^ and 2.4 × 10^−7^ M, respectively.

The performance characteristics for all these sensors are shown in Table 1. These results revealed that the sensors based on the use of *o*-NPOE as a solvent mediator displayed much better performance towards azide determination than other sensors based on DBS and DOP. Table 2 shows a comparison of the general performance of the proposed solid-contact azide sensors with those previously reported.

### 2.2. Robustness

The sensitivity of the proposed method to variations of experimental conditions (temperature, pH, and sample size) was tested. The ruggedness test was done using “Youden and Steiner partial factorial design” where eight replicate analyses were conducted, and three factors are varied and analyzed.

The pH effect on the potentiometric response of the proposed sensors was tested over a pH range of 2 to 11 with two fixed sodium azide concentrations (1.0 × 10^−4^ and 1.0 × 10^−3^ M). The potential response was pH independent in the pH range 5–10 and 6–9 for Nit-N_3_/POT and FePC/POT based membrane sensors, respectively. At pH > 10, the potential decreased probably due to competition of the OH^–^ with the N_3_^–^ anion. At pH < 5, a positive potential drift was noticed indication a decrease in azide concentration due to the formation and volatilization of hydrazoic acid (HN_3_) gas.

Variation of the concentration of azide samples over the range 10^−5^–10^−2^ M did not affect the accuracy by more than 1%. Change of the temperature of the test solution from 18–25 °C slightly affected the results. The simplest form of the Nernst equation is: *E* = *E*^o^-(0.055/n) log *c*. However, the 0.055/n part of the equation is a simplification of 2.303RT/nF. So, at 18 °C, 2.303*RT*/*F* = 0.053 volts and upon increasing the temperature to 25 °C, this value goes up to 0.055 volts.

The response time and stability of the proposed sensors were measured by recording the time required by the two sensors to reach a stable steady-state potential (within ± 0.3 mV). A response time of less than 10 s was obtained for all azide solutions in the linear calibration range of the two examined sensors. Potential stability was tested by following within-day repeatability and between-days reproducibility of the potentiometric response of the sensors. The potentials response remained constant within ± 0.3 mV for at least 30 min. The results obtained with 6 identical sensors, prepared and used over a period of 2 months showed a standard deviation not exceeding ± 1.1 mV without observing any considerable changes in the selectivity and response time.The reproducibility of the calibration slope was within ± 1.5 mV/decade over a period of 8 weeks (*n* = 6). After two months, the detection limit gradually changed about half an order of magnitude.

### 2.3. Sensors’ Selectivity

Potentiometric selectivity coefficients (*Kpot N_3_^−^*,*J*) of azide sensors based on nitron-azide (Nit-N_3_) and iron (II) phthalocyanine (FePC) plasticized with *o*-nitrophenyloctyl ether (*o*-NPOE) were evaluated using the modified separate solutions method (MSSM) [30]. The measured potentiometric selectivity coefficients of Nit-N_3_/POT and FePC/POT are given in Table 3. It can be seen that the selectivity coefficients of Nit-N_3_/POT based sensor towards different anions were in the order: Sal^–^ >I^–^ > ClO_4_^–^ > SCN^–^ > NO_3_^−^ > NO_2_^–^ > CH_3_COO^–^ > Cl^−^ > Br^−^ > SO_4_^2−^ > PO_4_^3−^. This order agreed fairly well with the classical Hofmeister series [1]. However, FePC/POT/based sensor, exhibited enhanced selectivity for azide ion with an anti-Hofmeister order. The selectivity coefficients were in the order: ClO_4_^–^ > I^–^ > Sal^–^ > NO_3_^–^ > SCN^–^ > CH_3_COO^–^ > Cl^–^ > Br^–^ > NO_2_^–^ > SO_4_^2−^ > PO_4_^3−^. This selectivity sequence is similar to that reported with some metallophthalocyanine based sensors of other species [24,31,32].

### 2.4. Electrochemical Impedance Spectroscopy (EIS) Measurements

**Figure 3:**
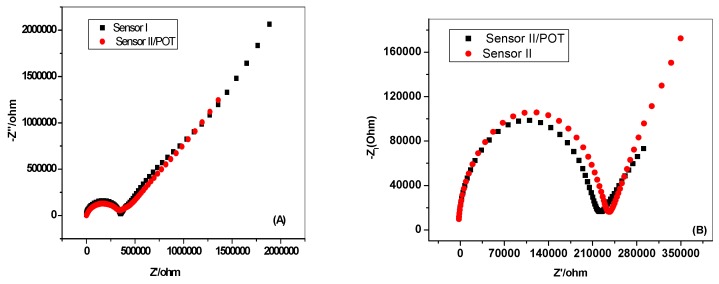
Electrochemical impedance spectroscopy (EIS) measurements of: (**A**) Nit-N_3_ and (**B**) FePC membrane based sensors.

EIS measurements were performed on the proposed sensors immersed in 10^−2^ M NaN_3_ solution. Examples of the impedance spectra of sensors with and without the solid contact (POT) were examined and the results are illustrated in Figure 3 for Nit-N_3_/POT, FePC/POT, Nit-N_3_ and FePC membranes. The high frequency semicircle can be related to the bulk impedance of the membranes. The bulk resistances (*R_b_*) of Nit-N_3_ and FePC membranes were found to be 0.37 and 0.24 MΩ, respectively. This slight difference in *R_b_* values may be attributed to the slight dissolution of POT in the membrane matrix. The time constant (*t_b_*) of the bulk process was calculated from the impedance spectra presented in Figure 3 using the frequency (*f_max_*) at the top of the high-frequency semicircle:

*t_b_* = (2*πf_max_)*^−1^ (1)

For Nit-N_3_ and FePC membrane-based sensors, the time constants were 0.32 and 0.25 ms, respectively. In the presence of POT, Nit-N_3_/POT and FePC/POT-based membrane sensors displayed smaller time constants of 0.28 and 0.20 ms, respectively. These results suggest that the bulk process of the FePC/POT membrane is faster than that of Nit-N_3_/POT membrane. In addition, the bulk process of the sensors became faster with the incorporation of POT.

As shown in Figure 3, the low frequency semicircle in both Nit-N_3_/POT and FePC/POT-based sensors is significantly smaller than for the same sensors without a POT layer. This indicates that the charge transfer impedance is decreased by incorporation of POT between the membrane and the solid electrical support. The double-layer capacitance (*C_dl_*) of Nit-N_3_/POT and FePC/POT membranes were *C_dl_* = 22.4 ± 0.7 and 13.8 ± 0.6 µF, respectively, compared with *C_dl_* = 4.7 ± 0.2 and 4.2 ± 0.4 µF for Nit-N_3_ and FePC membranes, respectively. This further confirm that the presence of POT as an ion-to-electron transducer significantly facilitate faster charge transport between the interfaces and offer more stable potential responses of the solid-contact ion selective sensor.

### 2.5. Chronopotentiometric Measurements

**Figure 4:**
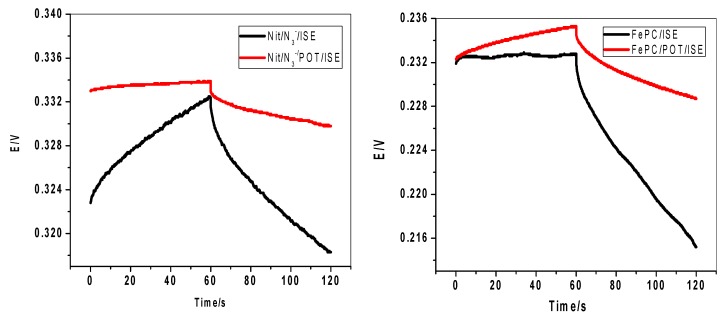
Measurements for azide membrane sensors with and without POT as a solid contact material.

The potential stability of the proposed sensors was evaluated by using constant current chronopotentiometric measurements [33]. In the absence of POT, Nit-N_3_ and FePC-based membrane sensors have large potential drift of 181.2 ± 3.1 and 226.0 ± 5.1 (*n* = 3) µV/s, respectively. However, much less potential drift of about 40.6 ± 1.9 and 69.2 ± 1.1 µV/s (*n* = 3) was noticed with Nit-N_3_/POT and Fe-PC/POT based membrane sensors, respectively. This declined potential is attributed to the high double layer capacitance of POT. The capacitances of the sensors were calculated and found to be 24.6 ± 0.8, 14.5 ± 0.7, 5.5 ± 0.3 and 4.42 ± 0.6 µF for Nit-N_3_/POT, FePC/POT, Nit-N_3_ and FePC-based membrane sensors, respectively. The data depicted in Figure 4 confirm a clear relationship between the potential stability (*ΔE/Δt*) or the capacitance (*C*) and the effect of POT solid contact. In addition, there is a good agreement between the results obtained by EIS and chronopotentiometry upon using POT confirming a high compatibility and adhesion between the solid conducting base and the polymeric membrane. This leads to extending the sensor response range, increasing the potential stability and improving the selectivity behavior.

### 2.6. Effect of Water Film Test of the Electrode Potential

**Figure 5:**
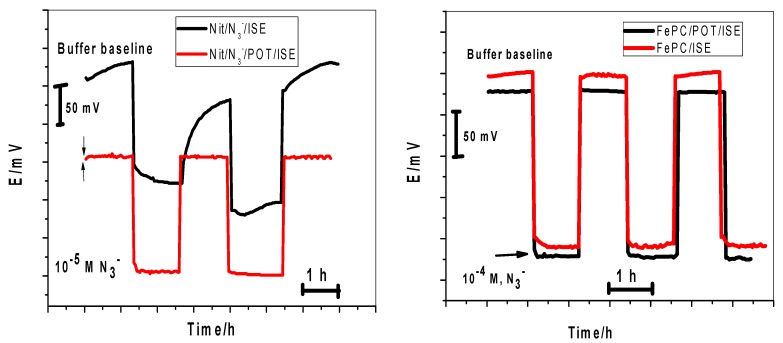
Effect of water-layer on azide membrane sensors with and without POT solid contact.

It is well documented that water film formation between polymeric sensing membranes and the solid conducting substrates causes significant potential drift and affect the sensitivity due to poor adhesion and delamination of the polymeric membrane. In the present work, a solid contact layer of poly(octylthiophene) (POT) was used between the solid conductor and polymeric sensing PVC membrane. The sensors were first conditioned in 10^−2^ M N_3_^−^ solution and then the sample was replaced with a solution of 10^−2^ M Na_2_SO_4_. A control experiment was performed by using Nit-N_3_ and FePC PVC membrane-based sensors without POT which are very close to the coated wire CWEs) configuration. As shown in Figure 5, positive EMF changes of ~145 and 200 mV are noted upon replacing 1.0 × 10^−5^ and 1.0 × 10^−4^ M N_3_^−^ solution with the electrolytic background solution of Nit-N_3_/POT and FePC/POT, respectively.

For Nit-N_3_ and FePC, a positive potential drift was noticed. This can be attributed to the formation of water layer between the sensing membranes and the conducting substrate. The stable response of Nit-N_3_/POT and FePC/POT-based sensors confirms the absence of a water layer, so the undesirable water layer can be successfully removed by the insertion of the high hydrophobic POT layer between the sensing membrane and the solid conducting substrate.

The long-term response of the proposed sensors was also tested. When not in use, these fabricated sensors were all conditioned in 1.0 × 10^−5^ M N_3_^−^ solution. Negligible change in the calibration slopes and detection limits was observed during 3 months. These results indicated the absence of the water films. The robust and reliable solid-contact N_3_^−^-ISEs are promising for applications in many fields of contemporary research.

### 2.7. Analytical Applications

Synthetic primer mixtures containing azide were prepared with azide content matched the real formulations and assessed by the proposed sensors. About 500 mg of KClO_3_ and 500 mg Sb_2_S_3_ (stibnite) were mixed with three different accurately measured amounts of sodium azide 1.0, 5.0 and 10.0 mg, respectively. Each mixture was dissolved in a 100 mL measuring flask and completed to the mark with deionized water. The azide contents of these mixtures were determined using the proposed sensors. As shown in Table 4, the analysis of mixtures containing 1.0, 5.0 and 10.0 mg azide showed average recoveries of 98–101%, 102.4–97.4% and 99.3–97.7% (*n* = 6) with the above tested concentrations, respectively.This confirmed the validity of the suggested method for the assessment of azide in complicated matrices.

## 3. Materials and Methods

### 3.1. Apparatus

Screen-printed azide PVC membrane sensors in conjunction with Ag/AgCl double junction reference electrode (model 90-02, Orion, Cambridge, MA, USA, USA) filled with 10% (w/v) K_2_SO_4_ in its outer compartment were used for measurement of azide. An Orion pH/meter (model SA 720, Cambridge, MA, USA) and a combination glass pH electrode (Schott blue line 25, Stuttgart, Germany) were used. The cell used for EMF measurements at ambient temperature was of the type: Ag/AgCl/KCl (10^−2^ M)/sample test solution//sensor membrane/POT/C.

Impedance and chronopotentiometric measurements were performed by applying a constant currents of ± 1 nA for 60s, on the screen-printed sensors in presence and absence of poly- (3-octylthiophene) (POT) by using an (Autolab Model 2000) potentiostat/galvanostat (Metrohom Instruments, Herisau, Switzerland). A reference electrode (Ag/AgCl (3 M KC1), and a platinum auxiliary electrode were used. The tested solution was 0.01 M N_3_^−^ ion. Chronopotentiometry was carried out on the proposed sensors by applying a constant currents of ± 1 nA for 60s, respectively. The impedance spectra were measured over the frequency range of 10 kHz to 0.1 Hz. The amplitude of the sinusoidal excitation signal was 50 mV. All experiments were performed at room temperature (23 ± 2 °C).

### 3.2. Reagents and Materials

All chemicals used were of analytical reagent grade unless stated otherwise, and doubly distilled water was used throughout. *o*-Nitrophenyloctyl ether (*o*-NPOE), bis(2-ethylhexyl)phthalate (DOP), dibutylsebacate (DBS), tris-hydroxymethylaminomethane (TRIS), tetradodecylammonium tetrakis(4-chlorophenyl) borate (ETH 500), poly(3-octylthiophene) (POT), tetrahydrofuran (THF), nitron (1,4-diphenylendoanilino-dihydrotriazole; 1,4-diphenyl-3-(phenylamino)-1*H*-1,2,4-triazole) and iron(II)phthalocyanine (FePC) were purchased from Sigma-Aldrich Chem. Co. (Steinheim, Germany). High relative molecular weight PVC was purchased from Fluka (Buchs, Switzerland). Sodium and potassium salts of all anions were purchased from Merck (Darmstadt, Germany). A stock solution of 0.1 M sodium azide was prepared in pre-boiled doubly distilled water. Working azide standards of different concentration in the range of 10^−2^ to 10^−9^ M were freshly prepared by stepwise dilutions. Tris buffer (0.1 M) of pH 7.0 was used to adjust the pH of all sample solutions. The ion activity coefficients were calculated according to the Debye–Hückel equation.

### 3.3. Nitron-azide Ion-pair Complex

A 10^−2^ M of nitron solution was prepared by dissolving 0.31 g of nitron in 100 mL of 20% acetic acid. A 20 mL portion of 10^−2^ M nitron solution was mixed with a 10 mL aliquot of 10^−2^ M NaN_3_. The solution was thoroughly mixed and stirred for 15 min. A brown precipitate was formed, filtered off, washed with bidistilled water, dried at room temperature and ground to a fine powder in agate mortar. Elemental analyses of the precipitate confirm the formation of 1:1 (azide: nitron) ion association complex.

### 3.4. Sensor Fabrication

Screen-printed ceramic carbon sensor substrate (96% alumina) was purchased from Gwent Electronic Materials Ltd. (Lancster, UK). A carbon electrode with an area of 3.1 mm and a diameter of 2 mm was used as a working electrode. The azide sensors were prepared by NaN_3_ through two steps; the first involved fabrication of the solid contact conducting substrate and the second dealt with screen printing of electroactive materials [34,35,36]. Poly(3-octylthiophene) (POT) in chloroform solution (2.0 mM) was successively drop-cast four times on the carbon-based contact and the chloroform solvent was allowed to evaporate after formation of each layer. The recognition membrane cocktail was prepared by mixing a 2.5 mg portion (2.5 wt %) of the ion sensing material (Nit-N_3_ or FePC), 2.0 mg (2.0% wt %) of ETH-500, 32.2 mg (32.2 wt %) of poly(vinylchloride) (PVC) and 63.3 mg (63.3 wt %) of *o*-NPOE and dissolving in 2 mL of THF. A 10 µL aliquot of the membrane cocktail was added dropwise over the POT layer through the circular orifice of the screen-printed wafer (SP) and left 4 h for drying at room temperature in the dark. A schematic representation of the solid-contact ion selective micro sensor is shown in Figure 1. The sensors were conditioned before use by soaking in a 1.0 × 10^−4^ M aqueous N_3_^–^ solution, and were stored in the same solution when not in use.

### 3.5. Sensor Calibration and Selectivity Measurements

The (FePC/POT) and (Nit-N_3_/POT)-based membrane sensors were calibrated by immersion, along with an Ag/AgCl reference electrode, in a 50-mL beaker containing 9.0 mL of 10^−2^ M Tris buffer solution of pH 7.0. Aliquots (1.0 mL) of standard sodium azide solution (1.0 × 10^−7^ to 1.0 × 10^−1^ M) were successively added with continuous stirring. The potential readout was recorded for each solution after stabilization to ± 0.5 mV (2 min). Calibration graphs connecting potential reading with logarithm azide concentrations were plotted and used for all subsequent unknown azide measurements. Selectivity coefficients were determined using the modified separate solutions method by recording separate calibration curves for all the interfering ions of interest [30]. The selectivity values were determined from the highest measured concentrations (0.1 M) with established formalisms.

## 4. Conclusions

Solid contact carbon screen-printed ceramic azide micro-sensors were developed, electrochemically characterized and used. These sensors are based on the utilization of ironII-phthalocyanine (Fe-PC) neutral carrier and nitron-azide ion-pair complex (Nit-N_3_^−^) as selective recognition receptors, POT as a solid contact material on a carbon printed ceramic substrate. The sensors displayed extended linear response range (1.0 × 10^−2^–1.0 × 10^−7^ M), low detection limit (1.0 × 10^−7^ and 7.7 × 10^−8^ M) and fast response time (< 10 s) for FePC/POT and Nit-N_3_/POT, membrane-based sensors, respectively. The potential sensitivity and stability of these sensors were tested by electrochemical impedance spectroscopy (EIS) and constant-current chronopotentiometry techniques. The proposed solid-contact azide-sensors were successfully used for trace azide quantification. The sensors offered good advantages over many of those previously described in terms of robustness, ease of fabrication, potential stability, selectivity, and accuracy. The sensors can be introduced in a flow system for contineous azide monitoring. No sample pretreatment is requried for azide analysis using these proposed sensors.

## Figures and Tables

**Table 1:** Response characteristics of azide membrane sensors in 0.01 M Tris buffer of pH 7.

Parameter	FePC/POT			Nit-N_3_/POT			
	*o-*NPOE	DOP	DBS	*o-*NPOE	DOP	DBS
Slope, (mV/decade)	−58.3 ± 0.9	−41.3 ± 0.6	−41.4 ± 0.2	−55.1 ± 0.7	−48.2 ± 0.6	−43.6 ± 0.5
Coefficient, (r) (n=3)	−0.998	−0.997	−0.999	−0.998	−0.997	0.999
Detection limit, (M)	1.0 × 10^−7^	4.3 × 10^−7^	7.2 × 10^−7^	7.7 × 10^−8^	2.1 × 10^−7^	2.4 × 10^−7^
Linear range, (M)	3.5 × 10^−7^–1.0 × 10^−2^	1.0 × 10^−6^–1.0 × 10^−2^	2.6 × 10^−6^–1.0 × 10^−2^	1.0 × 10^−7^–1.0 × 10^−2^	1.0 × 10^−6^–1.0 × 10^−2^	1.0 × 10^−6^–1.0 × 10^−2^
Response time, (s)	<10	<10	<10	<10	<10	<10
Working range, (pH)	5.0–10	5.0–10	5–10	6.0–9	6.0-9	6.0–9
Standard deviation, (%)	0.7	1.3	1.1	0.8	0.5	0.7
Accuracy, (%)	99.6	99.3	98.8	98.4	99.5	99.3
Precision, (%), Cv_w_(%)	1.1	1.2	1.7	0.7	1.0	1.2

**Table 2:** General features of some potentiometric azide membrane sensors based on different ionophores.

Ionophore	Slope, (mV/decade)	Linear Range, (M)	pH Range	Detection Limit, (M)	Interference	Ref.
Fe^III^- and Co^III^-complexes of 2,3,7,8,12,13,17,18-octakis (benzylthio)-5,10,15, 20-tetraazaporphyrin	−56.0	1.0 × 10^−5^– 3.5 × 10^−1^	2.3–6.4	1.0 × 10^−6^	SCN^−^, ClO_4_^−^, ClO_3_^−^, NO_3_^−^	[17]
Cyanoaquacobyric acid heptakis (2-phenylethyl ester)	−49.0	5.0 × 10^−5^– 1.0 × 10^−2^	6.0	-	NO_2_^−^	[18]
Substituted onium base salts	−57.6	1.0 × 10^−4^– 1.0 × 10^−1^	7.5–12.0	7.0 × 10^−5^	SO_4_^2−^, HCO_3_^−^, Cl^−^, H_2_PO_4_^−^	[19]
Fe^II^- and Ni^II^ –bathophenan- throlineazide ion-pair complexes	−29.2	8.9 × 10^−6^– 1.0 × 10^−1^	4.3-10.5	8.0 × 10^−7^	SCN^−^, S^2−^, NO_2_^−^, Cl^−^	[20]
Orion ammonium-sensitive gas probe model (95/12) with a Teflon semi-permeable membrane/Teflon membrane	−59.1	1.0 × 10^−4^– 1.0 × 10^−1^	1.0–3.5	3.5 × 10^−5^	SO_3_^2−^, NO_2_^−^, S^2−^, HCO_3_^−^, CH_3_COO^−^	[21]
Orion ammonium-sensitive electrode (model 95/12) with a polypropylene membrane	−58.8	1.0 × 10^−4^– 1.0 × 10^−1^	1.0–3.5	1.9 × 10^−5^	SO_3_^2−^, NO_2_^−^, S^2−^	[22]
Fe^III^-hydrotris-(3,5-dimethyl- pyrazolyl) borate acetylacetonate chloride	−59.4	6.3 × 10^−7^– 1.0 × 10^−2^	3.5–9.0	5.0 × 10^−7^	-	[23]
Fe^III^- Schiff base	−58.9	1.0 × 10^−6^– 5.0 × 10^−2^	4.3–10.2	8.8 × 10^−7^	ClO_3_^−^, IO_3_^−^, ClO_4_^−^, NO_2_^−^, NO_3_^−^, Cl^−^, I^−^	[24]
Mn(III)-porphyrin Co(II)-phthalocyanine	−56.3 -48.5	2.2 × 10^−5^– 1.0 × 10^−2^ 5.1 × 10^−5^ – 1.0 × 10^−2^	3.9–6.5 4.2–6.5	1.3 × 10^−5^ 1.7 × 10^−5^	I^−^, CN^−^ SO_3_^2−^	[25]
Mn^II^-[2-formylquinoline thiosemicarbazone] complex	−55.8	1.0×10^−5^–1.0 × 10^−2^	5.5–9.0	8.0 × 10^−6^	-	[26]
Fe-PC/POT Nit-N_3_/POT	−58.3 −55.1	3.5 × 10^−7^– 1.0 × 10^−2^ 3.5 × 10^−7^– 1.0 × 10^−2^	6.0–9.0 5.0–10.0	1.0 × 10^−7^ 7.7 × 10^−8^	- -	This work

**Table 3:** Potentiometric selectivity coefficients, *Log K^pot^_x,y_ , of the proposed screen-printed sensors.

Interfering ion	* Log K^pot^_x,y_	
	[FePC/POT	[Nit/N_3_/POT
PO_4_^3−^	−6.7 ± 0.2	−6.2 ± 0.5
Salicylate	−3.7 ± 0.4	−0.5 ± 0.1
NO_2_^−^	−5.27 ± 0.5	−3.1 ± 0.3
ClO_4_^−^	−3.3 ± 0.4	−0.8 ± 0.1
SCN^−^	−4.3 ± 0.7	−1.1 ± 0.1
I^−^	−3.5 ± 0.4	−0.6 ± 0.2
Cl^−^	−5.1 ± 0.3	−4.7 ± 0.3
Br^−^	−5.2 ± 0.1	−4.9 ± 0.3
SO_4_^2^^−^	−5.6 ± 0.4	−5.1 ± 0.4
CH_3_COO^−^	−4.9 ± 0.1	−4.2 ± 0.1
NO_3_^−^	−4.2 ± 0.3	−2.3 ± 0.5

* Mean of three measurements.

**Table 4:** Determination of azide in synthetic primer mixtures using the proposed azide sensors.

Sample	Taken, azide, mg/g	Azide, mg/g^a^			
		[Fe-PC/POT]	Recovery, %	[Nit-N_3_/POT]	Recovery, %
Mixture 1	1.0	0.98 ± 0.05	98.0	1.01 ± 0.2	101.0
Mixture 2	5.0	5.2 ± 0.4	102.4	4.87 ± 0.1	97.4
Mixture 3	10.0	9.93 ± 0.7	99.3	9.77 ± 0.3	97.7

^a^ Average of five measurements ± standard deviation.
